# Supplementary material for: 1.5 °C degrowth scenarios suggest the need for new mitigation pathways
Source: Nat Commun. 2021 May 11;12:2676. doi: 10.1038/s41467-021-22884-9 (PMC8113441; doi:10.1038/s41467-021-22884-9)
Supplement: Supplementary file 1 — Supplementary Information [file 41467_2021_22884_MOESM1_ESM.pdf]

Supplementary Information

1.5°C degrowth scenarios suggest the need for new mitigation pathways

Lorenz Keyßer[1,2] and Manfred Lenzen[1]

[1]ISA, School of Physics A28, The University of Sydney, Sydney, New South Wales, Australia.

[2]Institute for Environmental Decisions, Department of Environmental Systems Science, ETH Zürich, Zürich, Switzerland

Corresponding author: Lorenz T. Keyßer, email: lkeysser@student.ethz.ch

Supplementary Figures:

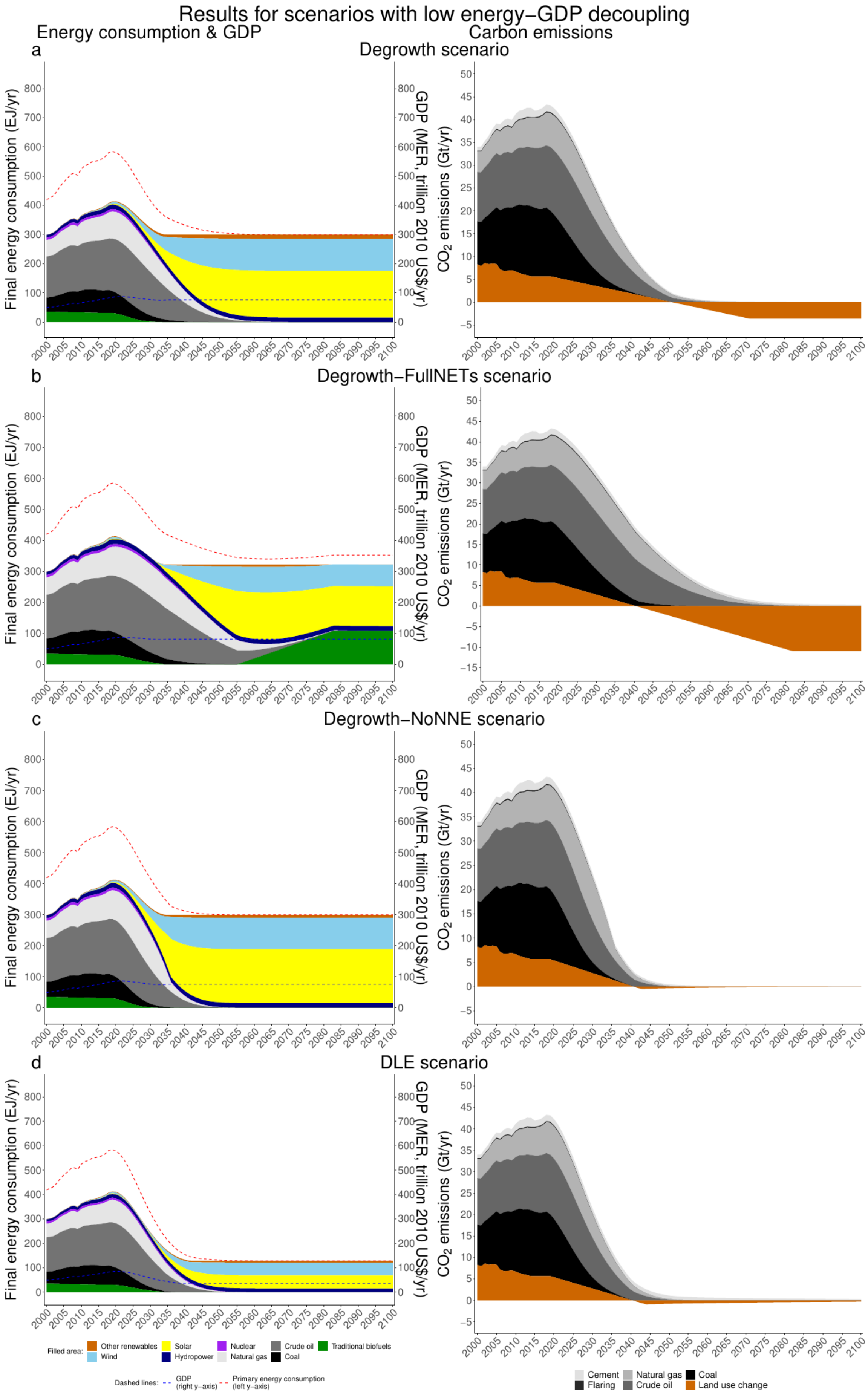

Figure S1. All 1.5°C scenarios with low energy-GDP decoupling (a-d). On the left, final energy consumption (in EJ, left axis), aggregate primary energy consumption (in EJ, red dashed line, left axis) and GDP (MER, in trillion 2010$, blue dashed line, right axis). On the right, carbon emissions (in GtCO2/yr).

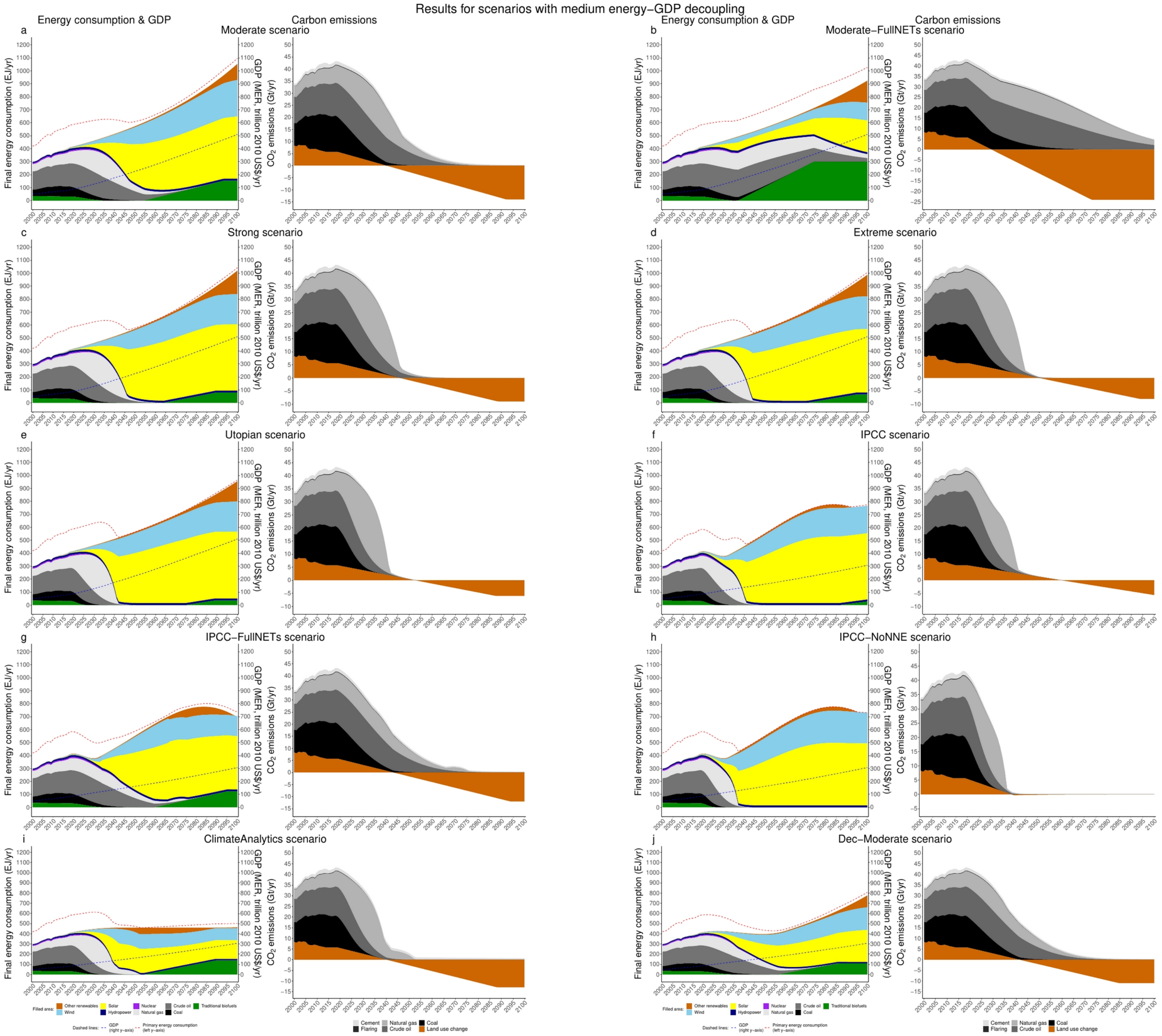

Figure S2. All 1.5°C scenarios with medium energy-GDP decoupling (a-j). On the left of each column, final energy consumption (in EJ, left axis), aggregate primary energy consumption (in EJ, red dashed line, left axis) and GDP (MER, in trillion 2010$, blue dashed line, right axis). On the right of each column, carbon emissions (in GtCO2/yr).

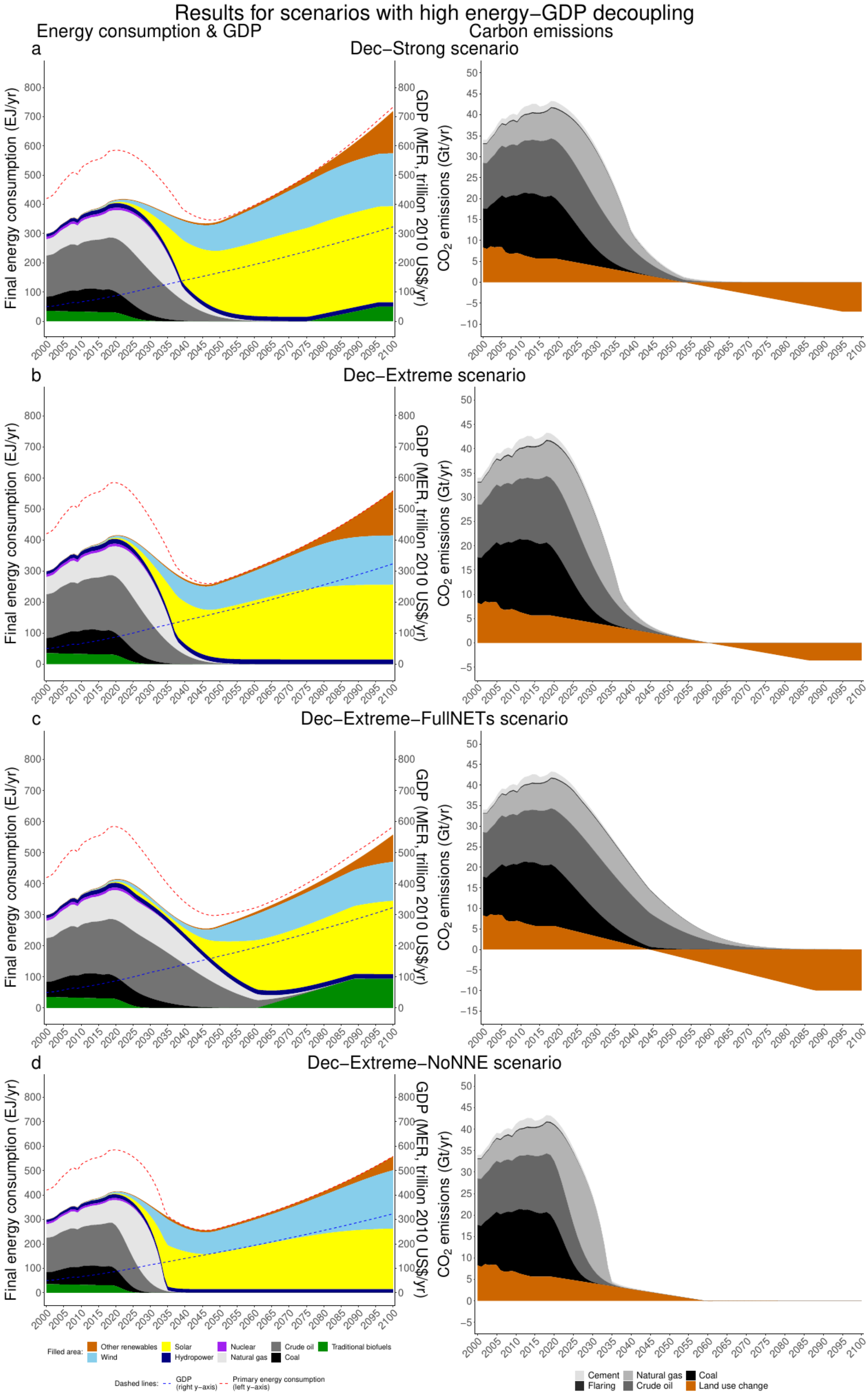
Results for scenarios with high energy−GDP decoupling
Energy consumption & GDP
Carbon emissions
a
Dec−Strong scenario
b
Dec−Extreme scenario
c
Dec−Extreme−FullNETs scenario
d
Dec−Extreme−NoNNE scenario
Final energy consumption (EJ/yr)
GDP (MER, trillion 2010 US$/yr)
CO2 emissions (Gt/yr)
Filled area:
Other renewables
Wind
Solar
Hydropower
Nuclear
Natural gas
Crude oil
Coal
Traditional biofuels
Dashed lines:
GDP (right y−axis)
Primary energy consumption (left y−axis)
Cement
Flaring
Natural gas
Crude oil
Coal
Land use change

Figure S3. All 1.5°C scenarios with high energy-GDP decoupling (a-d). On the left, final energy consumption (in EJ, left axis), aggregate primary energy consumption (in EJ, red dashed line, left axis) and GDP (MER, in trillion 2010$, blue dashed line, right axis). On the right, carbon emissions (in GtCO2/yr).

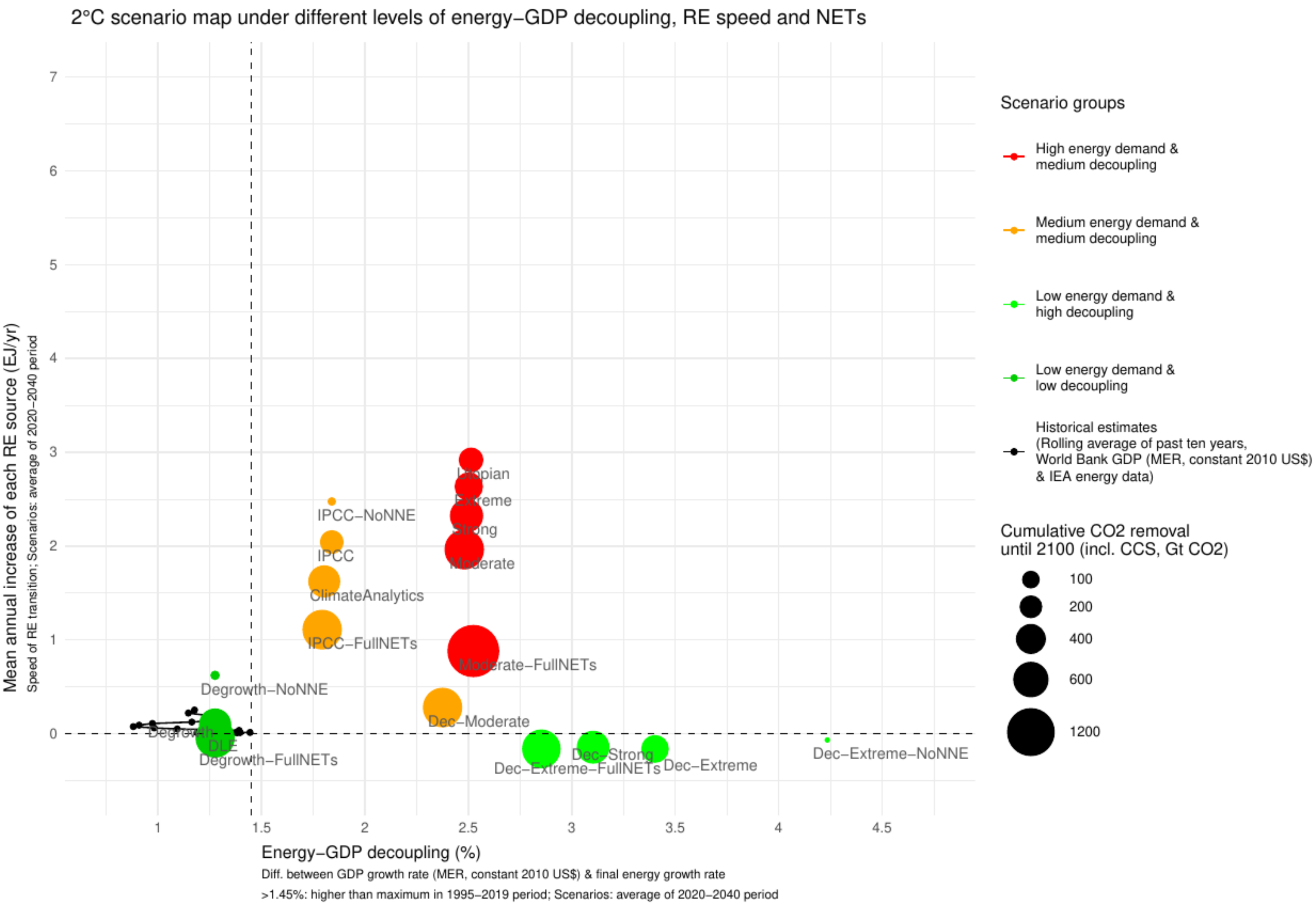

Figure S4. 2°C scenario map along the dimensions ‘speed of renewable energy transition’ (for the scenarios the 2020-2040 annual average growth in solar, wind and other renewables, in EJ/yr), ‘energy-GDP decoupling’ (for the scenarios the 2020-2040 average difference between GDP growth rate and final energy growth rate, in %) and cumulative CO_2 removal, including CCS ($GtCO_2$). Historical data points are the rolling averages of the past ten years (e.g. for the 1995 point the period 1986-1995) of the respective indicators. We excluded their labels here for readability, but they are the same as in Figure 5 in the article. GDP data (MER) is taken from the World Bank and the scenario parameters can be found in the tables below.

Supplementary Tables:

Table S1. Parameters for 2°C pathways with low energy-GDP decoupling. Carrier-dependent annual change rate increments δ_c and seed values $\gamma_c(t=2019)$ for annual rates of change as in Eq 1; constant change rates β as in Eq 4; constant annual increments λ as in Eq 5 in Methodology.

Low energy-GDP decoupling					
	Annual change rate increments δ_c (%)				Seed value (%)
Carrier	Degrowth	Degrowth-FullNETs	Degrowth-NoNNE	DLE	$\gamma_c(t=2019)$
Coal	-0.125	-0.1	-0.217	-0.8	-2.0
Crude oil	-0.125	-0.1	-0.217	-0.8	1.8
Natural gas	-0.125	-0.1	-0.217	-0.9	2.8
Nuclear	-1.0	-0.5	-1.0	-0.5	1.5
Traditional biofuels	-2.0	-1.0	-2.0	-1.0	-0.5
Hydro-electricity	0.0	0.0	0.0	0.0	0.0
Solar PV	-0.65	-0.75	-0.35	-5.0	28.8
Wind	-0.25	-0.35	0.05	-6.0	15.9
Other renewables	0.3	0.2	0.6	-0.5	0.5
Total FE demand	-0.9	-0.9	-0.9	-1.3	1.0
	Constant change rates β (%)				
Cement & flaring	-5.0	-2.0	-5.0	-2.0	
	Constant annual increments λ (Mt CO_2/a)				
Forestry & land use	-300	-376	-260	-265	
	Maximum rate of negative emissions (Gt CO_2/a)				
Forestry & land use	-11	-18.0	0.0	0.0	
	Maximum CCS share of energy from coal and natural gas (%)				
Coal & gas	0.0	0.0	0.0	0.0	0.0
	Linear increase rate of CCS (%/a)				
Coal & gas	0.0	0.0	0.0	0.0	0.0

Table S2. Parameters for 2°C pathways with medium energy-GDP decoupling (1). Carrier-dependent annual change rate increments δ_c and seed values $\gamma_c(t=2019)$ for annual rates of change as in Eq 1; constant change rates β as in Eq 4; constant annual increments λ as in Eq 5 in Methodology.

Medium energy-GDP decoupling (1)						
	Annual change rate increments δ_c (%)					Seed value (%)
Carrier	Moderate	Moderate-FullNETs	Strong	Extreme	Utopian	$\gamma_c(t=2019)$
Coal	-0.1	-0.06	-0.125	-0.15	-0.175	-2.0
Crude oil	-0.1	-0.06	-0.125	-0.15	-0.175	1.8
Natural gas	-0.1	-0.06	-0.125	-0.15	-0.175	2.8
Nuclear	-0.5	-0.5	-1.0	-1.5	-2.0	1.5
Traditional biofuels	-1.0	-1.0	-2.0	-3.0	-5.0	-0.5
Hydro-electricity	0.0	0.0	0.0	0.0	0.0	0.0
Solar PV	-0.75	-0.75	-0.65	-0.55	-0.4	28.8
Wind	-0.3	-0.35	-0.25	-0.15	-0.05	15.9
Other renewables	0.2	0.2	0.3	0.4	0.5	0.5
Total FE demand	0.004	0.0	0.003	0.002	0.001	1.0
	Constant change rates β (%)					
Cement & flaring	-2.0	-2.0	-5.0	-7.5	-7.5	
	Constant annual increments λ (Mt CO_2/a)					
Forestry & land use	-319	-555	-240	-177	-140	
	Maximum rate of negative emissions in Gt CO_2/a					
Forestry & land use	-16.0	-24.0	-10.0	-8.0	-6.0	
	Maximum CCS share of energy from coal and natural gas (%)					
Coal & gas	30.0	30.0	35.0	40.0	45.0	0.0
	Linear increase rate of CCS (%/a)					
Coal & gas	1.33	1.33	1.58	1.83	2.08	0.0

Table S3. Parameters for 2°C pathways with medium energy-GDP decoupling (2). Carrier-dependent annual change rate increments δ_c and seed values $\gamma_c(t=2019)$ for annual rates of change as in Eq 1; constant change rates β as in Eq 4; constant annual increments λ as in Eq 5 in Methodology.

Medium energy-GDP decoupling (2)						
	Annual change rate increments δ_c (%)					Seed value (%)
Carrier	IPCC	IPCC-FullNETs	IPCC-NoNNE	Climate Analytics	Dec-Moderate	$\gamma_c(t=2019)$
Coal	-0.175	-0.1	-0.214	-0.136	-0.1	-2.0
Crude oil	-0.175	-0.1	-0.214	-0.136	-0.1	1.8
Natural gas	-0.175	-0.1	-0.214	-0.136	-0.1	2.8
Nuclear	-2.0	-0.5	-2.0	-1.0	-0.5	1.5
Traditional biofuels	-5.0	-1.0	-5.0	-2.0	-1.0	-0.5
Hydro-electricity	0.0	0.0	0.0	0.0	0.0	0.0
Solar PV	-0.40	-0.73	-0.40	-0.40	-0.75	28.8
Wind	-0.05	-0.35	-0.05	-0.05	-0.30	15.9
Other renewables	0.5	0.2	0.5	0.6	0.2	0.5
Total FE demand	-0.7	-0.7	-0.7	-0.05	-0.15	1.0
	Constant change rates β (%)					
Cement & flaring	-7.5	-2.0	-7.5	-1.0	-2.0	
	Constant annual increments λ (Mt CO_2/a)					
Forestry & land use	-136	-286	-285	-270	-316	
	Maximum rate of negative emissions in Gt CO_2/a					
Forestry & land use	-3.6	-16.0	0.0	-13.0	-16.0	
	Maximum CCS share of energy from coal and natural gas (%)					
Coal & gas	45.0	45.0	0.0	0.0	30.0	0.0
	Linear increase rate of CCS (%/a)					
Coal & gas	2.08	2.08	0.0	0.0	1.33	0.0

Table S4. Parameters for 2°C pathways with high energy-GDP decoupling. Carrier-dependent annual change rate increments δ_c and seed values $\gamma_c(t=2019)$ for annual rates of change as in Eq 1; constant change rates β as in Eq 4; constant annual increments λ as in Eq 5 in Methodology.

High energy-GDP decoupling					
	Annual change rate increments δ_c (%)				Seed value (%)
Carrier	Dec-Strong	Dec-Extreme	Dec-Extreme-FullNETs	Dec-Extreme-NoNNE	$\gamma_c(t=2019)$
Coal	-0.125	-0.15	-0.1	-0.236	-2.0
Crude oil	-0.125	-0.15	-0.1	-0.236	1.8
Natural gas	-0.125	-0.15	-0.1	-0.236	2.8
Nuclear	-1.0	-1.5	-1.5	-2.0	1.5
Traditional biofuels	-2.0	-3.0	-3.0	-5.0	-0.5
Hydro-electricity	0.0	0.0	0.0	0.0	0.0
Solar PV	-0.65	-0.55	-0.75	0.25	28.8
Wind	-0.25	-0.15	-0.35	0.65	15.9
Other renewables	0.3	0.4	0.2	1.1	0.5
Total PE demand	-0.3	-0.5	-0.5	-0.5	1.0
	Constant change rates β (%)				
Cement & flaring	-5.0	-7.5	-7.5	-7.5	
	Constant annual increments λ (Mt CO_2/a)				
Forestry & land use	-240	-177	-295	-141	
	Maximum rate of negative emissions in Gt CO_2/a				
Forestry & land use	-9.0	-6.0	-13.0	0.0	
	Maximum CCS share of energy from coal and natural gas (%)				
Coal & gas	35.0	40.0	40.0	0.0	0.0
	Linear increase rate of CCS (%/a)				
Coal & gas	1.58	1.83	1.83	0.0	0.0
