# Supplementary material for: 1.5 °C degrowth scenarios suggest the need for new mitigation pathways
Source: Nat Commun. 2021 May 11;12:2676. doi: 10.1038/s41467-021-22884-9 (PMC8113441; doi:10.1038/s41467-021-22884-9)
Supplement: Supplementary file 3 — Description of Additional Supplementary Files [file 41467_2021_22884_MOESM3_ESM.pdf]

Description of Additional Supplementary Files

File Name: Supplementary Data 1
Description: A full version of our simplified quantitative representation of the fuel-energy-emissions nexus for 1.5°C scenarios, as described in the Methods. Together with the information provided in the Methods and Supplementary Information, all our scenarios can be reproduced and other ones explored.
