# Supplementary material for: 1.5 °C degrowth scenarios suggest the need for new mitigation pathways
Source: Nat Commun. 2021 May 11;12:2676. doi: 10.1038/s41467-021-22884-9 (PMC8113441; doi:10.1038/s41467-021-22884-9)
Supplement: Supplementary file 5 — Reporting Summary [file 41467_2021_22884_MOESM5_ESM.pdf]

nature research

Corresponding author(s): Lorenz T. Keyßer
Last updated by author(s): Mar 4, 2021

Reporting Summary

Nature Research wishes to improve the reproducibility of the work that we publish. This form provides structure for consistency and transparency in reporting. For further information on Nature Research policies, see our Editorial Policies and the Editorial Policy Checklist.

Statistics

For all statistical analyses, confirm that the following items are present in the figure legend, table legend, main text, or Methods section.

n/a	Confirmed	
☒	☐	The exact sample size (*n*) for each experimental group/condition, given as a discrete number and unit of measurement
☒	☐	A statement on whether measurements were taken from distinct samples or whether the same sample was measured repeatedly
☒	☐	The statistical test(s) used AND whether they are one- or two-sided<br>*Only common tests should be described solely by name; describe more complex techniques in the Methods section.*
☒	☐	A description of all covariates tested
☒	☐	A description of any assumptions or corrections, such as tests of normality and adjustment for multiple comparisons
☒	☐	A full description of the statistical parameters including central tendency (e.g. means) or other basic estimates (e.g. regression coefficient) AND variation (e.g. standard deviation) or associated estimates of uncertainty (e.g. confidence intervals)
☒	☐	For null hypothesis testing, the test statistic (e.g. *F*, *t*, *r*) with confidence intervals, effect sizes, degrees of freedom and *P* value noted<br>*Give P values as exact values whenever suitable.*
☒	☐	For Bayesian analysis, information on the choice of priors and Markov chain Monte Carlo settings
☒	☐	For hierarchical and complex designs, identification of the appropriate level for tests and full reporting of outcomes
☒	☐	Estimates of effect sizes (e.g. Cohen's *d*, Pearson's *r*), indicating how they were calculated

Our web collection on statistics for biologists contains articles on many of the points above.

Software and code

Policy information about availability of computer code

Data collection	The model projections of the global energy mix as well as the CO2 emission were made using Microsoft Excel Version 15.14.
Data analysis	The resulting data was analyzed and visualized using R-Studio Version 1.3.1093 and R Version 3.6.3.

For manuscripts utilizing custom algorithms or software that are central to the research but not yet described in published literature, software must be made available to editors and reviewers. We strongly encourage code deposition in a community repository (e.g. GitHub). See the Nature Research guidelines for submitting code & software for further information.

Data

Policy information about availability of data

All manuscripts must include a data availability statement. This statement should provide the following information, where applicable:

- Accession codes, unique identifiers, or web links for publicly available datasets
- A list of figures that have associated raw data
- A description of any restrictions on data availability

All relevant data underpinning our modelling are cited throughout the study and Methods. We further deposit a full version of our model, as described in the Methods, in the Supplementary Information as a Supplementary Data file.

Field-specific reporting

Ecological, evolutionary & environmental sciences study design

All studies must disclose on these points even when the disclosure is negative.

Study description	Building on historical data for the fuel-energy-emissions nexus, we project 18 1.5°C scenarios for global final and primary energy use as well as carbon emissions until 2100 under varying assumptions regarding energy use, speed of renewable energy expansion and negative emission technologies. The scenarios are constructed such to fit the range reported in the IPCC Special Report on 1.5°C, while additionally expanding the range to include degrowth scenarios as well as scenarios without any net negative emissions. This is done to compare IPCC scenario archetypes on the one hand and degrowth scenarios, which are currently neglected by most IAM modelling as well as the IPCC, on the other, in order to assess their relative risks for feasibility and sustainability. This risk assessment is conducted with extensive reference to the literature and by comparing the scenarios regarding indicator values drawn from the modelling.
Research sample	We investigate 18 scenarios: four pathways with low energy-GDP decoupling (the consumption-driven degrowth pathways: ‘Degrowth’, ‘Degrowth-FullNETs’, ‘Degrowth-NoNNE’ and ‘DLE’ (Decent Living Energy)), ten scenarios with medium energy-GDP decoupling (the technology-driven scenarios: ‘Moderate’, ‘Moderate-FullNETs’, ‘Strong’, ‘Extreme’, ‘Utopian’, ‘IPCC’, ‘IPCC-FullNETs’, ‘IPCC-NoNNE’, ‘ClimateAnalytics’ and ‘Dec-Moderate’), as well as four technology-driven pathways with high energy-GDP decoupling (called ‘Dec-Strong’, ‘Dec-Extreme’, ‘Dec-Extreme-FullNETs’, ‘Dec-Extreme-NoNNE’). This pwathway sample was chosen in order to broadly cover the range in the IPCC SR1.5 and to additionally cover degrowth scenarios and scenarios without net negative emissions.<br><br>Here 'X-FullNETs' stands for a scenario version of its standard run 'X' with lower speed of renewable energy expansion and a higher amount of negative emission technologies, while 'NoNNE' stands for a scenario version without any net negative emissions, but higher renewable speeds.
Sampling strategy	The scenarios were developed such to represent the range of the primary energy use pathways reported in the IPCC Special Report on 1.5°C and especially the four archetype scenarios described in the report. We describe the respective broad equivalent within the IPCC in Table 1 with in the paper. For instance our 'IPCC' scenarios closely track the median primary energy trajectory of the IPCC, while our degrowth scenarios best represent the Low Energy Demand scenario by Grubler et al. (2018).<br><br>The scenarios further differ in terms of speed of renewable energy expansion and negative emission technologies, including carbon capture and storage applied to coal and gas. These values were adjusted to make the scenarios meet the 1.5°C target by 2100, but also to show a variety of values on the speed of renewable expansion and negative emissions and thus be broadly representative of the ranges reported by the IPCC. CCS values were also adopted from the IPCC, as described in detail in the Methodology.
Data collection	Prof. Manfred Lenzen developed the quantitative model and collected the initial data from the literature. Lorenz Keyßer and Prof. Manfred Lenzen designed the scenarios following the above procedure (see also our Methodology for more details) as well as analyzed and visualized the resulting data using the mentioned programes.
Timing and spatial scale	Our analysis covers global historical data from 1985 to 2019 on final and primary energy use and its composition as well as global carbon emissions from fossil fuels and land use. Our model projects these categories until 2100, which is the commonly used end date for climate mitigation scenarios in the IPCC.
Data exclusions	No data points were excluded from this analysis.
Reproducibility	All experiments can be reproduced using our procedure, data and assumptions as stated in our Methodology and Supplementary Material Additionally, the model itself can be found in the Supplementary Data.
Randomization	Randomization is not relevant here because the purpose of this study is to deliberately compare IPCC Special Report on 1.5°C emission scenarios with degrowth scenario regarding certain key risk indicators for feasibility and sustainability.
Blinding	Again, this was was not relevant to our study, due to the stated purpose and Methodology.

Did the study involve field work? ☐ Yes ☒ No

Reporting for specific materials, systems and methods

We require information from authors about some types of materials, experimental systems and methods used in many studies. Here, indicate whether each material, system or method listed is relevant to your study. If you are not sure if a list item applies to your research, read the appropriate section before selecting a response.

Materials & experimental systems

n/a	Involved in the study	
☒	☐	Antibodies
☒	☐	Eukaryotic cell lines
☒	☐	Palaeontology and archaeology
☒	☐	Animals and other organisms
☒	☐	Human research participants
☒	☐	Clinical data
☒	☐	Dual use research of concern

Methods

n/a	Involved in the study	
☒	☐	ChIP-seq
☒	☐	Flow cytometry
☒	☐	MRI-based neuroimaging
